# Association between the onset age of puberty and parental height

**DOI:** 10.1371/journal.pone.0211334

**Published:** 2019-01-25

**Authors:** Yehuda Limony, Slawomir Koziel, Michael Friger

**Affiliations:** 1 Pediatric Endocrinology Unit, Faculty of Health Sciences, Ben-Gurion University of the Negev, Clalit Health Services, Beer-Sheva, Israel; 2 Department of Anthropology, Hirszfeld Institute of Immunology and Experimental Therapy, Polish Academy of Sciences, Wrocław, Poland; 3 Department of Public Health, Faculty of Health Sciences, Ben-Gurion University of the Negev, Beer-Sheva, Israel; CUNY, UNITED STATES

## Abstract

**Background:**

The onset age of physiological puberty is greatly variable. This variability has been attributed to environmental factors and to genetic factors although a very little is explained by genome-wide associations studies. Previously, we reported the existence of an association between the onset age of puberty and final height. It is known that final height is associated with parental height (specifically, with the "target height"). We hypothesized that the variability of the onset age of puberty contributes to the attainment of a final height which is similar to the target height. We hypothesized that whenever a child's height-percentile differs from the target height percentile (we called this difference the "height gap"), the onset of puberty is advanced or delayed so that they are closer or even equal at the end of pubertal growth. The association between height gap and onset age of puberty was investigated in the reported study.

**Methods:**

The study is an observational retrospective study on growth during puberty in 170 Israeli (60 girls) and 335 Polish children (162 girls). Anthropometric measurements were analyzed by multivariable linear regression with the onset age of the pubertal growth spurt (PGS) as the dependent variable, and two independent variables "height gap" and body mass index (BMI)—both standardized.

**Results:**

The adjusted coefficient of determination (adj R^2^) between the onset age of the PGS and the two independent variables was 0.69 (Israeli girls), 0.50 (Israeli boys, BMI excluded), 0.25 (Polish girls) and 0.13 (Polish boys). A prediction model for the onset age of puberty is presented.

**Conclusions:**

The association between the "height gap" and the onset age of puberty suggests that the variability of this age is part of the targeted process of statural growth. The proposed model may explain idiopathic cases of precocious and delayed puberty.

## Introduction

The onset age of physiologic puberty is greatly variable, and this variability has been attributed to genetic and environmental factors [1–3]. Although the early onset of puberty has been linked to high values of body mass index (BMI)) and tall stature in childhood [4–6], a model, which explains the physiologic variability of onset age of puberty, is still lacking.

In the USA, the range of variation in the onset age of puberty in the nineties was greater than that in the sixties mainly because of early onset age of puberty [7–10]. The causes of this increased range are unknown although obesity was suspected.

Early and delayed onsets of puberty are generally normal physiologic events. At the extremes of the normal range of onset age of puberty (mean ± 2 years), the possibility of pathology is increased. Although the probability of pathology (a brain tumor for example) is rare, its health impact may be great. Furthermore, early puberty may result in a short adult stature which may impact wellness [11]. Early puberty is associated also with cardiovascular disease [12] that is probably independent of obesity [13], breast cancer [14], and endometrial and ovarian cancers [15]. A delayed puberty in females is also associated with an increased risk of bone fractures throughout life [16]. These health impacts may concern children, parents, and physicians. In order to exclude underlying brain pathology, children, whose onset age of puberty is close to the extremes of the normal range, are referred for diagnostic testing. These tests, which often include magnetic resonance imaging, are expensive and cause discomfort to children and their families. The usual result of these tests is a diagnosis of idiopathic precocious or delayed puberty. Although it was recommended to lower the threshold of the physiologic age of onset of puberty, this recommendation has been questioned by many pediatric endocrinologists and pediatricians in the USA because of a possible misdiagnosis of pathologic precocious puberty [17]. Accordingly, improving our knowledge about the physiologic timing of puberty would help to better diagnose the pathologic puberty and obviate many diagnostic procedures.

Previously, we reported a correlation between final height standard deviation score (SDS) and both the age and height-SDS at the onset of puberty [11]. Children grow toward a final height that is within a range called "the target range". The midpoint of this target range is called "the target height" and can be calculated from the average of the parents' height-SDS. It is known that children usually maintain the same height percentile (and the same height-SDS) during their pre-pubertal growth. Since the height-SDS of a child typically doesn't change during childhood, the attainment of a target height percentile that is different from childhood's height percentile is dependent on the variability of the onset age of puberty. We hypothesized that the variability in the onset age of puberty reflects the variability in target height and the variability in statural growth during childhood. We also hypothesized that the onset age of puberty is correlated with the difference between the height-SDS of the child and child’s target height-SDS. We also incorporated the BMI-SDS or BMI-percentile as an independent variable to the regression analysis because it has been reported to influence the onset age of puberty [4,18]. We tested our hypothesis using data from two groups of normal adolescents.

## Methods

We conducted an observational retrospective study on growth and puberty in two separate groups of Polish and Israeli children. The Polish group comprised 335 children (162 girls) from nine randomly selected Wroclaw elementary schools. They were followed prospectively at annual intervals from age 8 years until age 18 years (boys) and 17 years (girls) from 1961 to 1972 (The Wroclaw Growth Study) [11, 19–20].

The Israeli group comprised 170 children (60 girls) who had been referred to an endocrinology clinic in southern Israel from 2004 to 2015 for the following reasons: a normal but below average stature, a short stature, an early puberty, or a late puberty (histograms of the parameters may be found in supporting information). Children with a chronic disease, a genetic syndrome, or any pathology were excluded from the study. It is emphasized that all participants had no pathology related to their height or puberty. The children and their parents were measured using a wall-mounted stadiometer and a calibrated scale was used to measure children's weight. The children were measured periodically at different intervals of time (from 4 to 18 months) according to clinical needs. In order to precisely determine the onset age of the pubertal growth spurt (PGS), only children that had at least two height measurements before the onset of the PGS and at least two height measurements after the age of peak height velocity were included in the study. S1 Dataset. Participants' parameters.

The onset age of the PGS was computed for every child using Karlberg’s infancy-childhood-puberty (ICP) model [21]. A computer program automatically fitted the ICP model to the individual height records and generated the onset age of the PGS for each child by subtracting two years from the age at the peak height velocity (PHV). Height measurements (cm) were converted to SDS using equations of the ICP model [22]. The height-SDS at the onset of the PGS was calculated using an ICP-based interpolation between two adjacent measurements: before and after the onset age of the PGS. Weight at the onset of the spurt was calculated by linear interpolation of two measurements adjacent to the onset age of the PGS. In order to improve the accuracy of the determination of the onset age of the PGS, only measurements that were made within one year from each other were used in the interpolation. Data from 26 Israeli girls were excluded from the interpolation because this criterion was not met. The mean values of mid-parental height and pre-pubertal height of the girls that were excluded were not different statistically from the corresponding values of the girls who were included in the study. BMI was calculated using the weight and height at the onset age of the PGS. Some weight data of Israeli boys were missing (unknown cause) and some weight data comprised measurements that were not made at the same time as the height measurements. As a result, the weight data of only 38 Israeli boys were included in the regression analysis. The mean and SD values of the parameters that were to be used in the regression analyses from the group of 110 boys and the group of 38 boys were not significantly different.

Since including the BMI (percentile or SDS) as an independent variable didn’t change the calculated correlation for other variables in the Israeli boys, this parameter was omitted from their final analysis.

The BMI data were converted to BMI percentiles and SDS (Z score) using the CDC tables and equations [23].

The mid-parental height-SDS was derived from and expressed as the average between the father's height-SDS and the mother's height-SDS.

The difference between the child's height at the onset of the PGS and the mid-parental height (both expressed in SDS) was termed as the "height gap".

All data were analyzed using a computerized statistical software program (SPSS version 17.0 for Windows; SPSS, Chicago, IL, USA).

A two-tailed t-test was used to compare the mean values of the various study parameters and statistical significance was set at 5%.

The study in Israel was approved (no. 0157-16-COM1) and informed consent was exempted by the Meir Medical Center’s review board (Helsinki Committee). The study was retrospective and the data were fully anonymized before the authors had access to them.

The Wroclaw Growth Study was approved by the Polish Academy of Sciences and details of the study were provided to the parents of each participating child. A parent or both parents voluntarily came to the Institute of Anthropology with the child for examination in April/May 1961, and then at annual intervals. Parental participation in the examinations was accepted as implicit informed consent. Polish law at the time of the study did not require written consent of parents for children to participate in growth-related studies. Participation in the project was voluntary and children were free to withdraw at any time. The study was retrospective and the data were fully anonymized before the authors had access to them.

## Results

Mean values of the onset age of the PGS, BMI (percentile and SDS), mid-parental height (SDS), pre-pubertal height (SDS) and the height gap are presented in Table 1.

**Table 1:** Mean values (95% confidence interval) of the parameters used in the regression analysis.

	Onset age of the PGS	BMI percentile	BMI-SDS	Mid-parental height-SDS	Pre-pubertal height-SDS	Height gap
Israeli girls (n = 60)	9.70 (9.24–10.16)	47.3 (37.7–57.0)	-0.14 (-0.52–0.24)	-1.29 (-1.51- -1.06)	-0.92 (-1.30- -0.54)	0.37 (0.09–0.65)
Polish girls (n = 162)	10.02 (9.90–10.13)	32.20 (28.28–36.13)	-0.58 (-0.70- -0.45)	-1.24 (-1.36- -1.13)	-0.89 (-1.04- -0.74)	0.35 (0.23–0.47)
Israeli boys (n = 110)	12.55 (12.30–12.80)	49.02 (n = 38) (37.05–61.00)	0.04 (n = 38) (-0.42–0.50)	-1.46 (-1.66- -1.27)	-1.70 (-1.95- -1.45)	-0.24 (-0.40- -0.07)
Polish boys (n = 173)	11.96 (11.80–12.12)	39.87 (36.10–43.64)	-0.32 (-0.44- -0.21)	-1.21 (-1.32- -1.10)	-0.63 (-0.78- -0.47)	0.58 (0.43–0.73)

The onset age of puberty was negatively correlated with anthropometric parameters of the child and his/her parents. The correlation in the Israeli group was higher than that in the Polish group (Tables 2 and 3). According to the results of the regression analysis when the BMI remains constant, an increase of 1 SDS in the height gap is associated with advancement of the onset age of puberty by 0.9 years (Israeli girls), 1.07 years (Israeli boys), 0.25 years (Polish girls), and 0.34 years (Polish boys). Likewise, a decrease in height gap of 1 SDS is associated with a delay in the onset age of puberty of the same magnitude.

**Table 2:** Results of the regression analysis of the onset age of puberty with the height gap and the BMI percentiles.

Israeli girls (n = 60) R^2^ = 0.70 Adj R^2^ = 0.69	Independent variables	coefficient	SE	standardized coefficient	p-value
	Height gap (SDS)	-0.90	0.156	-0.556	<0.001
	BMI (percentile)	-0.017	0.004	-0.358	<0.001
	Intercept	10.84			
Polish girls (n = 162) R^2^ = 0.26 Adj R^2^ = 0.25					
	Height gap (SDS)	-0.25	0.068	-0.266	<0.001
	BMI (percentile)	-0.011	0.002	-0.372	<0.001
	Intercept	10.47			
Israeli boys (n = 110) R^2^ = 0.50 Adj R^2^ = 0.50					
	Height gap (SDS)	-1.07	0.103	-0.7068	<0.001
	intercept	12.29			
Polish boys (n = 173) R^2^ = 0.14 Adj R^2^ = 0.13					
	Height gap (SDS)	-0.34	0.079	-0.312	<0.001
	BMI (percentile)	-0.007	0.003	-0.162	0.026
	Intercept	12.43			

**Table 3:** Results of the regression analysis of the onset age of puberty with the height gap and the BMI-SDS.

Israeli girls (n = 60) R^2^ = 0.69 Adj R^2^ = 0.68	Independent variables	coefficient	SE	Standardized coefficient	p-value
	Height gap (SDS)	-0.96	0.153	-0.589	<0.001
	BMI (SDS)	-0.389	0.114	-0.322	<0.001
	Intercept	9.99			
Polish girls (n = 162) R^2^ = 0.25 Adj R^2^ = 0.24					
	Height gap (SDS)	-0.25	0.068	-0.269	<0.001
	BMI (SDS)	-0.332	0.064	-0.364	<0.001
	Intercept	9.921			
Israeli boys (n = 110) R^2^ = 0.50 Adj R^2^ = 0.50					
	Height gap (SDS)	-1.07	0.103	-0.7068	<0.001
	Intercept	12.29			
Polish boys (n = 173) R^2^ = 0.14 Adj R^2^ = 0.13					
	Height gap (SDS)	-0.34	0.078	-0.311	<0.001
	BMI (SDS)	-0.229	0.099	-0.167	0.021
	Intercept	12.08			

The correlations between each independent parameter (Height-gap and BMI) and the onset age of puberty as the dependent parameters are presented in graphs (1 to 2) in the appendix (S1, S2, S3 and S4 Graphs).

The coefficients in Tables 2 and 3 can be used for predictive modeling of the onset age of puberty in a population.

For example, the model for Israeli girls would be as follows:

Onset age of puberty = 10.84–0.90 * height gap—0.017 * BMI percentile

Substituting the BMI percentile with the BMI-SDS, the following equation results:

Onset age of puberty = 9.99–0.96 * height gap—0.389 * BMI SDS

The residuals in all regression analyses were normally distributed (residual plots no. 3, 4 in the appendix S1, S2, S3 and S4 Graphs) and the calculated variance inflation factor was low for all predictor variables (less than 1.8) which mean that the predictor variables have a very low level of collinearity.

In all groups except for the group of Polish girls, using height-SDS of the child resulted in lower correlation than that obtained by height gap. Specifically, the value of the adjusted coefficient of determination (adj R^2^) for the (a) Polish boys was 0.10, (b) Polish girls was 0.29, (c) Israeli girls was 0.61, and (d) Israeli boys was 0.24.

Converting the BMI data to Z scores (SDS) instead of percentiles did not change significantly the results of the various correlations (Table 3).

## Discussion

This study was done on two groups of children which differed in their geographic locations and in the timeliness of data collection: the Israeli children were measured between the years 2004 and 2015 and the Polish children were measured between 1961 and 1972. Since the study tested a hypothesis which is related to the physiology of puberty, this difference between the two groups constitutes the study’s strength. Environmental influences on the onset age of puberty may change rapidly but physiologic factors are unlikely to change, if at all, during a few decades. In this study, we describe an association between the "height gap" and the onset age of puberty in two groups of children in whom height measurements were done longitudinally during two different periods in two different countries.

In studies on the timing of puberty, the age of menarche is usually chosen because the specific date can be identified. Unlike prospective studies, this date in retrospective studies relies on memory and therefore is less reliable. In this study, we used another sign of puberty: the onset of the PGS which occurs two years before its peak [21]. The time of menarche and PGS are correlated: menarche occurs about 1.3 years after the peak of the PGS (correlation between 0.8 and 0.9) [24]. The correlation between the times of menarche and the PGS indicates the existence of hormonal coupling. This coupling and the precise determination of the onset of the PGS makes the onset of the PGS an ideal marker in retrospective studies on the onset age of puberty and constitutes the strength of this study.

The onset age of the PGS was calculated by subtracting two years from the age at the PHV which was generated by the computer program. We also visually inspected the growth velocity graphs which were generated by the computer to make sure that a PGS which deviates grossly from the ICP model (in amplitude or time duration) would be excluded from the study. A PGS with a shortened duration between its onset age and age at PHV would have been discovered by this inspection. None of the children had abnormal PGS. We cannot exclude the possibility that a very mild shortening in PGS duration was concealed but this concealing may have a very little impact on the study’s results.

Much has been written on the association between obesity and early puberty in girls [4, 18, 25–31]. The results of this study also reveal the existence of an association between high values of BMI percentiles and early puberty in girls. In boys, the reports are inconsistent: some report a positive association between obesity and early puberty [18, 26, 28–33], some report a negative association [34–36] and some report no association [37, 38]. This inconsistency in boys exists also in this study: we found no association between the BMI percentile and the onset age of puberty in the Israeli boys and a low association between the BMI percentile and the onset age of puberty in the Polish boys. Only three children (one girl) had a BMI greater than 2 SDS. In view of the recent obesity epidemic and its possible role in advancing the onset age of puberty, the lack of any obese children in our groups is a weakness of this study.

**Fig 1:**
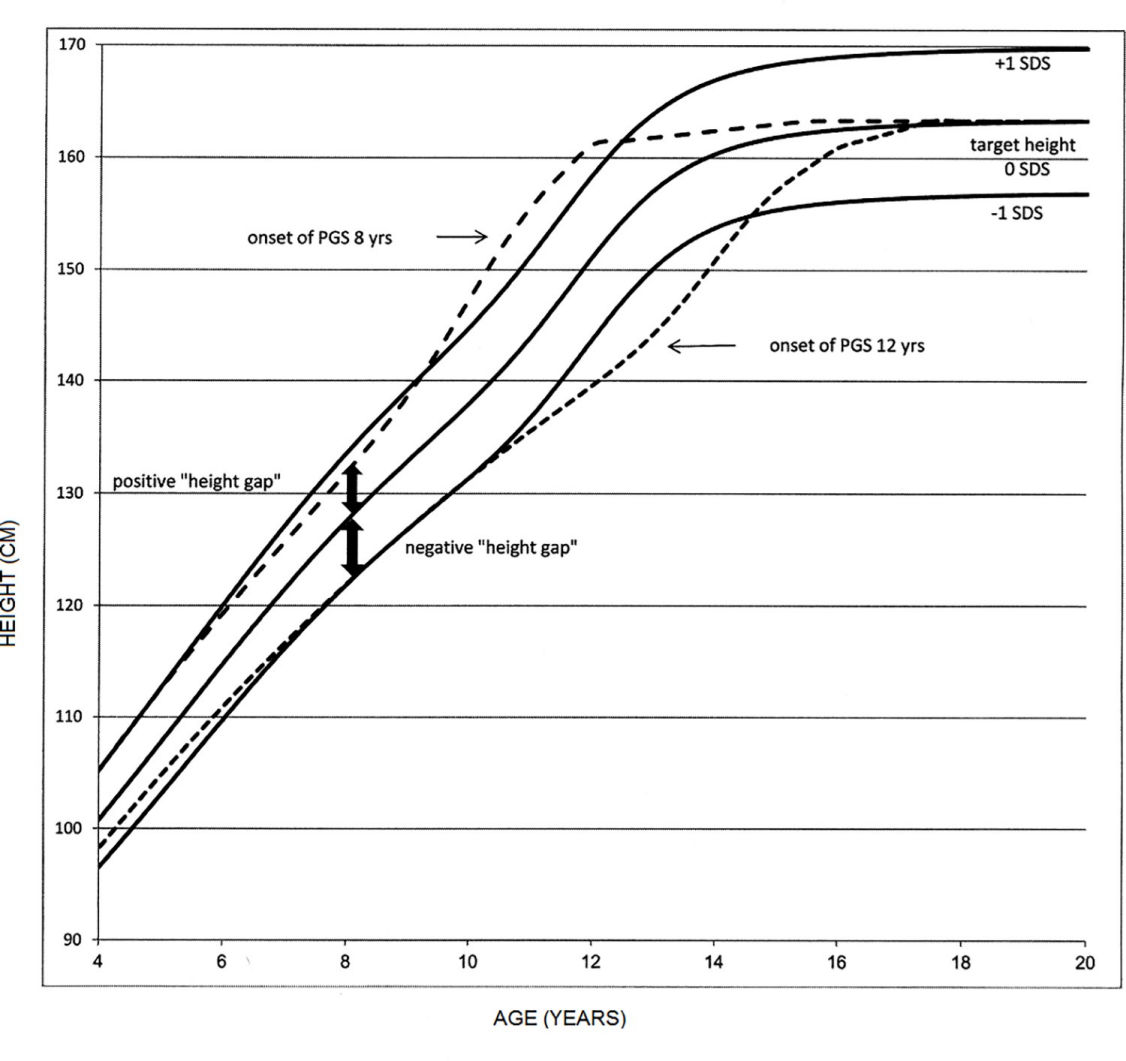
"Height gap" and the age of onset of the pubertal growth spurt. In this example, the target height is set to 0 SDS. The lines of 0, +1 and -1 SDS were generated using the Z scores of the heights of girls from the CDC 2000 tables [23]. The lines that describe the growth of girls whose onset of the PGS occurred at 8 years and 12 years were generated using the equations of the ICP model [21]. The "height gap" is the difference between the pre-pubertal child's height and his/her target height both of which are expressed in SDS. As is shown in this example, the positive "height gap" is associated with an early PGS and the negative "height gap" is associated with a delayed PGS.

In this study, we introduce a new term "height gap". In our previous study [11], we reported that the final height-SDS is shorter than the onset height-SDS for a PGS that starts earlier than average and vice versa. Statural growth in children is a targeted process: it ends within a "target range" that its midpoint is termed the "target height". In this study, we hypothesized that the variation in the timing of the PGS is part of this targeting: children who grow in childhood along a height percentile which is higher than their target height percentile will start their PGS earlier than average and children who grow along a height percentile which is shorter than their target height will start their PGS later than average (Fig 1). The result would be narrowing of the height gap and a final height which is closer to the target height. We also hypothesized that the greater is a positive "height gap", the earlier will puberty start and vice versa, the greater is a negative "height gap", the later will puberty start. In this study, we assumed that the target height equals the mid-parental height as suggested by Tanner [39]. When analyzing a population with a known change in adult height, it is advised to replace the value of the mid-parental height with a "corrected mid-parental height" value that takes into account the secular trend [40]. This replacement will not change the correlation of the regression nor will it change the coefficients of the parameters. It will only change the intercept and the predictions will become more accurate. Practically, the change in the predicted onset age is small because the secular trend in one generation is also very small. For example, a +0.29 SDS change in the target height of girls results in a delay to the onset age of puberty of 0.07 years and a +0.43 SDS change in the target height of boys results in a delay to the onset age of puberty of 0.15 years when the Polish prediction equation is used. The identical changes in the target height of the Israeli children result in a change of 0.26 years in girls and 0.46 years in boys.

The regression analysis confirmed our hypotheses on the existence of a correlation between a) the height gap and BMI and b) the onset age of puberty in both groups. Interestingly, the degree of the correlation was greater in the Israeli group than in the Polish group.

It is possible that other factors in the Polish group reduced the impact of the height gap and BMI on the onset age of puberty. One possible factor that may affect the height gap is probably the time when the height gap is created. The height gap may be created during gestation, in infancy or in childhood. A child born small for gestational age (SGA) and doesn't catch up in growth during infancy may have a negative height gap. When a negative height gap is created after birth and in infancy, it is called constitutional delay of growth and puberty (CDGP). Both children (the SGA and the CDGP) may have the same negative height gap but differ in the time when this height gap is created. It is known that CDGP is associated with a delayed puberty and there are studies [41, 42] that report that SGA children have a high risk for earlier puberty. In other words, the same height gap created in different periods of growth may have different or even opposite effects on the onset age of puberty. Unfortunately, we couldn't check this possibility because we have no growth data from the infancy period of the two groups.

Our finding of the association between height gap and age at onset of puberty in two very different groups suggests that this association may exist in other populations. More studies in different ethnic groups are necessary to confirm the consistency of our results.

Our hypothesis on the role of height in the onset age of puberty is not new. One of the proposed explanations on the variability of the age of puberty is a hypothetical "somatometer", an innate brain mechanism, which tracks growth. This "somatometer" monitors somatic development and activates the gonadotropin-releasing hormone pulse generator to initiate puberty in synchrony with the target adult height [43]. If a somatometer does exist, we posit that the height gap is included in its elaborate "algorithm".

The correlation between the height gap and the onset of puberty may contribute to our current understanding of idiopathic cases of early/precocious or late puberty in the following situations:

- Constitutional advancement of growth is characterized by an early and sometimes precocious puberty. This type of growth refers to children whose lengths at birth are average but whose height percentiles at the age of 2–3 years are above the average. Since most of these children have parents with average adult height, a positive height gap is created with early puberty [5].
- Constitutional delay in growth and puberty: this type of growth is characterized by a delayed puberty. Growth attenuation takes place in the first years of life (until 3–5 years). A negative height gap is created because these children usually have averaged-statured parents [44].
- The early onset age of puberty in the USA: advancement of puberty was seen in obese Afro-Americans who were described as being taller than their peers before, but not after, puberty [9]. Therefore, it is probable that a positive height gap, in addition to obesity, is associated with their early puberty.
- Positive secular trends in height are greater at the age of two years than in adulthood (and no trend is seen after the age of two years) [45]. It is possible that this positive height gap at the age of two years advances their onset age of puberty and reduces the impact of the trend on adult height.
- Adopted children: precocious puberty has been observed in adopted children when they relocate from a developing country to a developed one. The results of a study on adopted children in Denmark revealed that the risk for precocious puberty was 10- to 20-fold greater in the adopted children than in the native Danish children. A positive height gap which is created from a combination of (a) having short stature parents (usually living in poverty) and (b) an accelerated growth after adoption may explain this advancement in the onset age of puberty [46–47].

A predictive model for the onset age of puberty may facilitate the distinction between physiologic and pathologic puberty. We assume that the greater the time difference between the actual onset age and the predicted onset age, the higher is the probability of pathology. Consider an Israeli girl whose height is +1.21 SDS, a mid-parental height of -1 SDS (height gap = +2.21), and a BMI in the 80^th^ percentile. According to the proposed model, the expected start of her PGS is 7.5 years. Consider a girl with the identical height and BMI and taller parents (the mid-parental height is +0.55 SDS), her height gap is only +0.5 and the predicted onset age of puberty is 8.9 years. According to our predictive model, a PGS which starts at the age of 7.5 years can probably be considered physiologic in the first girl but should be suspected as being pathologic in the second girl.

The height SDS of children is stable after infancy until the onset of puberty. A significant change in height SDS during childhood (for example more than 0.3 SDS over one year) must be evaluated to exclude pathology or to diagnose the start of the pubertal spurt (in the case of accelerated growth). Therefore, the height gap should not be calculated whenever there is unstable height growth.

We found the coefficients for the height gap different in the two cohorts of children. Therefore, we recommend that reliable and accurate models should probably be constructed separately for different ethnic groups and different environments because the effect of the height gap may change in different populations and environments.

In conclusion, we found an association between the onset age of puberty and two childhood parameters: body fat as represented by a standardized BMI (percentile or SDS) and a child's height relative to parent's height as represented by the "height gap". The association between the onset age of puberty and the height gap may indicate that the variability of the onset age of puberty contributes to the targeting process of statural growth. Our findings raise anew the theory of a "somatometer" which synchronizes the onset age of puberty with the attainment of target height. It is probable that the variability of the onset age of puberty is the result of an interaction between physiologic factors which regulate attainment of the target height and non-physiologic factors, such as the environment, socioeconomic status and genetic mutations, which can interfere with the physiology of puberty and the attainment of target height. In view of our results, the effect of the "height gap" may be considered physiologic because it contributes to the attainment of target height and that of the BMI an interfering factor. Since many factors are probably responsible for the variability of the onset age of puberty, an accurate predictive model must include as many influencing factors as possible and should always include the physiologic factors. In view of our findings, we suggest that the "height gap" should be included in any model for predicting the onset age of puberty.

## Supporting information

S1 Dataset Participants' parameters. (XLSX)

S1 Graphs Israeli girls. (DOCX)

S2 Graphs Israeli boys. (DOCX)

S3 Graphs Polish girls. (DOCX)

S4 Graphs Polish boys. (DOCX)

## References

[1] Cousminer DL, Berry DJ, Timpson NJ, Ang W, Thiering E, Byrne EM, et al Genome-wide association and longitudinal analyses reveal genetic loci linking pubertal height growth, pubertal timing and childhood adiposity. Hum Mol Genet. 2013;22: 2735–2747. 10.1093/hmg/ddt104 23449627 PMC3674797

[2] Euling SY, Selevan SG, Pescovitz OH, Skakkebaek NE. Role of Environmental Factors in the Timing of Puberty. Pediatrics. 2008;121: S167–S171. 10.1542/peds.2007-1813C 18245510

[3] Toppari J, Juul A. Trends in puberty timing in humans and environmental modifiers. Mol Cell Endocrinol. 2010;324: 39–44. 10.1016/j.mce.2010.03.011 20298746

[4] Rosenfield RL, Lipton RB, Drum ML. Thelarche, Pubarche, and Menarche Attainment in Children With Normal and Elevated Body Mass Index. Pediatrics. 2009;123: 84–88. 10.1542/peds.2008-0146 19117864

[5] Papadimitriou A, Nicolaidou P, Fretzayas A, Chrousos GP. Constitutional Advancement of Growth, a.k.a. Early Growth Acceleration, Predicts Early Puberty and Childhood Obesity. J Clin Endocrinol Metab. 2010;95: 4535–4541. 10.1210/jc.2010-0895 20610589

[6] Lundeen EA, Norris SA, Martorell R, Suchdev PS, Mehta NK, Richter LM, et al Early Life Growth Predicts Pubertal Development in South African Adolescents. J Nutr. 2016;146: 622–629. 10.3945/jn.115.222000 26843589 PMC4763484

[7] Marshall WA, Tanner JM. Variations in the Pattern of Pubertal Changes in Boys. Arch Dis Child. 1970;45: 13–23. 10.1136/adc.45.239.13 5440182 PMC2020414

[8] Marshall WA, Tanner JM. Variations in pattern of pubertal changes in girls. Arch Dis Child. 1969;44: 291–303. 10.1136/adc.44.235.291 5785179 PMC2020314

[9] Herman-Giddens ME, Slora EJ, Wasserman RC, Bourdony CJ, Bhapkar MV, Koch GG, et al Secondary sexual characteristics and menses in young girls seen in office practice: a study from the Pediatric Research in Office Settings network. Pediatrics. 1997;99: 505–512. 9093289 10.1542/peds.99.4.505

[10] Herman-Giddens ME, Steffes J, Harris D, Slora E, Hussey M, Dowshen SA, et al Secondary Sexual Characteristics in Boys: Data From the Pediatric Research in Office Settings Network. Pediatrics. 2012;130: e1058–e1068. 10.1542/peds.2011-3291 23085608

[11] Limony Y, Kozieł S, Friger M. Age of onset of a normally timed pubertal growth spurt affects the final height of children. Pediatr Res. 2015;78: 351–355. 10.1038/pr.2015.104 26020145

[12] Qiu C, Chen H, Wen J, Zhu P, Lin F, Huang B, et al Associations Between Age at Menarche and Menopause With Cardiovascular Disease, Diabetes, and Osteoporosis in Chinese Women. The Journal of Clinical Endocrinology & Metabolism. 2013;98: 1612–1621. 10.1210/jc.2012-2919 23471979

[13] Dreyfus J, Jacobs DR, Mueller N, Schreiner PJ, Moran A, Carnethon MR, et al Age at Menarche and Cardiometabolic Risk in Adulthood: The Coronary Artery Risk Development in Young Adults Study. The Journal of Pediatrics. 2015;167 10.1016/j.jpeds.2015.04.032 25962931 PMC4516565

[14] Bodicoat DH, Schoemaker MJ, Jones ME, Mcfadden E, Griffin J, Ashworth A, et al Timing of pubertal stages and breast cancer risk: the Breakthrough Generations Study. Breast Cancer Research. 2014;16 10.1186/bcr3613 24495528 PMC3978643

[15] Fujita M, Tase T, Kakugawa Y, Hoshi S, Nishino Y, Nagase S, et al Smoking, Earlier Menarche and Low Parity as Independent Risk Factors for Gynecologic Cancers in Japanese: A Case-Control Study. The Tohoku Journal of Experimental Medicine. 2008;216: 297–307. 10.1620/tjem.216.297 19060444

[16] Bonjour J-P, Chevalley T. Pubertal Timing, Bone Acquisition, and Risk of Fracture Throughout Life. Endocrine Reviews. 2014;35: 820–847. 10.1210/er.2014-1007 25153348

[17] Rosenfield RL, Bachrach LK, Chernausek SD, Gertner JM, Gottschalk M, Hardin DS, et al Current Age of Onset of Puberty. Pediatrics. 2000;106: 622–623. 10.1542/peds.106.3.622 11012339

[18] Holmgren A, Niklasson A, Nierop AF, Gelander L, Aronson AS, Sjöberg A, et al Pubertal height gain is inversely related to peak BMI in childhood. Pediatric Research. 2016;81: 448–454. 10.1038/pr.2016.253 27861464

[19] Bielicki T, Waliszko A. Wrocław growth study part I: females. Stud Phys Anthropol 1975;2:53–83.

[20] Waliszko A, Jedliǹska W. Wrocław growth study part II: males. Stud Phys Anthropol 1976;3:27–48.

[21] Karlberg J. A Biologically-Oriented Mathematical Model (ICP) for Human Growth. Acta Paediatr. 1989;78: 70–94. 10.1111/j.1651-2227.1989.tb11199.x 2801108

[22] Karlberg J, Kwan C-W, Gelander L, Albertsson-Wikland K. Pubertal Growth Assessment. Hormone Research in Paediatrics. 2003;60: 27–35. 10.1159/000071223 12955015

[23] Growth Charts—2000 CDC Growth Charts—United States [internet].Cdc.gov. Available from: https://www.cdc.gov/growthcharts/cdc_charts.htm Cited 01 June 2018.

[24] Falkner F, Tanner JM, editors. Human growth: a comprehensive treatise Vol. 2: Postnatal growth. Neurobiology. 2. ed New York: Plenum Press; 1986.

[25] Addo OY, Miller BS, Lee PA, Hediger ML, Himes JH. Age at hormonal onset of puberty based on luteinizing hormone, inhibin B and body composition in preadolescent US girls. Pediatr Res. 2014;76: 564–570. 10.1038/pr.2014.131 25192395

[26] He Q, Karlberg J. BMI in Childhood and Its Association with Height Gain, Timing of Puberty, and Final Height. Pediatr Res. 2001;49: 244–251. 10.1203/00006450-200102000-00019 11158521

[27] Kaplowitz PB. Link Between Body Fat and the Timing of Puberty. Pediatrics. 2008;121: S208–S217. 10.1542/peds.2007-1813F 18245513

[28] Lundeen EA, Norris SA, Martorell R, Suchdev PS, Mehta NK, Richter LM, et al Early Life Growth Predicts Pubertal Development in South African Adolescents. The Journal of Nutrition. 2016;146: 622–629. 10.3945/jn.115.222000 26843589 PMC4763484

[29] Leonibus CD, Marcovecchio ML, Chiavaroli V, Giorgis TD, Chiarelli F, Mohn A. Timing of puberty and physical growth in obese children: a longitudinal study in boys and girls. Pediatric Obesity. 2013;9: 292–299. 10.1111/j.2047-6310.2013.00176.x 23713062

[30] Marceau K, Ram N, Houts RM, Grimm KJ, Susman EJ. Individual differences in boys and girls timing and tempo of puberty: Modeling development with nonlinear growth models. Developmental Psychology. 2011;47: 1389–1409. 10.1037/a0023838 21639623 PMC3928626

[31] Aksglaede L, Juul A, Olsen LW, Sørensen TIA. Age at Puberty and the Emerging Obesity Epidemic. PLoS ONE. 2009;4 10.1371/journal.pone.0008450 20041184 PMC2793517

[32] Sandhu J, Ben-Shlomo Y, Cole TJ, Holly J, Smith GD. The impact of childhood body mass index on timing of puberty, adult stature and obesity: a follow-up study based on adolescent anthropometry recorded at Christs Hospital (1936–1964). International Journal of Obesity. 2005;30: 14–22. 10.1038/sj.ijo.0803156 16344844

[33] Song Y, Ma J, Wang H-J, Wang Z, Lau PWC, Agardh A. Age at spermarche: 15-year trend and its association with body mass index in Chinese school-aged boys. Pediatric Obesity. 2015;11: 369–374. 10.1111/ijpo.12073 26403948

[34] Reinehr T, Bosse C, Lass N, Rothermel J, Knop C, Roth CL. Effect of Weight Loss on Puberty Onset in Overweight Children. The Journal of Pediatrics. 2017;184 10.1016/j.jpeds.2017.01.066 28238482

[35] Lee JM, Wasserman R, Kaciroti N, Gebremariam A, Steffes J, Dowshen S, et al Timing of Puberty in Overweight Versus Obese Boys. Pediatrics. 2016;137 10.1542/peds.2015-0164 26817933

[36] Lee JM, Kaciroti N, Appugliese D, Corwyn RF, Bradley RH, Lumeng JC. Body Mass Index and Timing of Pubertal Initiation in Boys. Archives of Pediatrics & Adolescent Medicine. 2010;164 10.1001/archpediatrics.2009.258 20124142 PMC4172573

[37] Buyken AE, Karaolis-Danckert N, Remer T. Association of prepubertal body composition in healthy girls and boys with the timing of early and late pubertal markers. The American Journal of Clinical Nutrition. 2008;89: 221–230. 10.3945/ajcn.2008.26733 19056586

[38] He F, Guan P, Liu Q, Crabtree D, Peng L, Wang H. The relationship between obesity and body compositions with respect to the timing of puberty in Chongqing adolescents: a cross-sectional study. BMC Public Health. 2017;17 10.1186/s12889-017-4681-1 28821292 PMC5563015

[39] Falkner F, Tanner JM editors. Human growth: a comprehensive treatise Vol. 3: Methodology. Ecological, genetic, and nutritional effects on growth. 2. ed New York: Plenum Press; 1986.

[40] Luo ZC, Albertsson-Wikland K, Karlberg J. Target Height as Predicted by Parental Heights in a Population-Based Study. Pediatric Research. 1998;44: 563–571. 10.1203/00006450-199810000-00016 9773847

[41] Verkauskiene R, Petraitiene I, Wikland KA. Puberty in Children Born Small for Gestational Age. Hormone Research in Paediatrics. 2013;80: 69–77. 10.1159/000353759 23899516

[42] Roth CL, Sathyanarayana S. Mechanisms affecting neuroendocrine and epigenetic regulation of body weight and onset of puberty: Potential implications in the child born small for gestational age (SGA). Reviews in Endocrine and Metabolic Disorders. 2012;13: 129–140. 10.1007/s11154-012-9212-x 22415297

[43] Plant TM. Neuroendocrine control of the onset of puberty. Front Neuroendocrinol. 2015;38: 73–88. 10.1016/j.yfrne.2015.04.002 25913220 PMC4457677

[44] Crowne EC, Shalet SM, Wallace WH, Eminson DM, Price DA. Final height in boys with untreated constitutional delay in growth and puberty. Arch Dis Child. 1990;65: 1109–1112. 10.1136/adc.65.10.1109 2248500 PMC1792322

[45] Cole T. The secular trend in human physical growth: a biological view. Economics & Human Biology. 2003;1: 161–168. 10.1016/s1570-677x(02)00033-3 15463971

[46] Teilmann G, Pedersen CB, Skakkebaek NE, Jensen TK. Increased Risk of Precocious Puberty in Internationally Adopted Children in Denmark. Pediatrics. 2006;118: e391–e399. 10.1542/peds.2005-2939 16882780

[47] Virdis R, Street ME, Zampolli M, Radetti G, Pezzini B, Benelli M, et al Precocious puberty in girls adopted from developing countries. Arch Dis Child. 1998;78: 152–154. 10.1136/adc.78.2.152 9579158 PMC1717454

