# Supplementary material for: Association between the onset age of puberty and parental height
Source: PLoS One. 2019 Jan 25;14(1):e0211334. doi: 10.1371/journal.pone.0211334 (PMC6347184; doi:10.1371/journal.pone.0211334)
Supplement: S4 Graphs — (DOCX) [file pone.0211334.s005.docx]

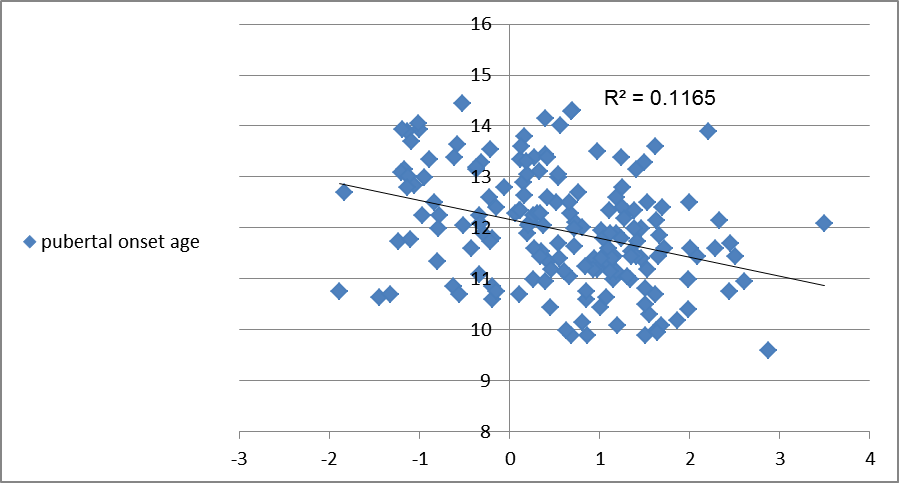


**Fig 1. Polish boys: Pubertal onset age vs height gap.**

**
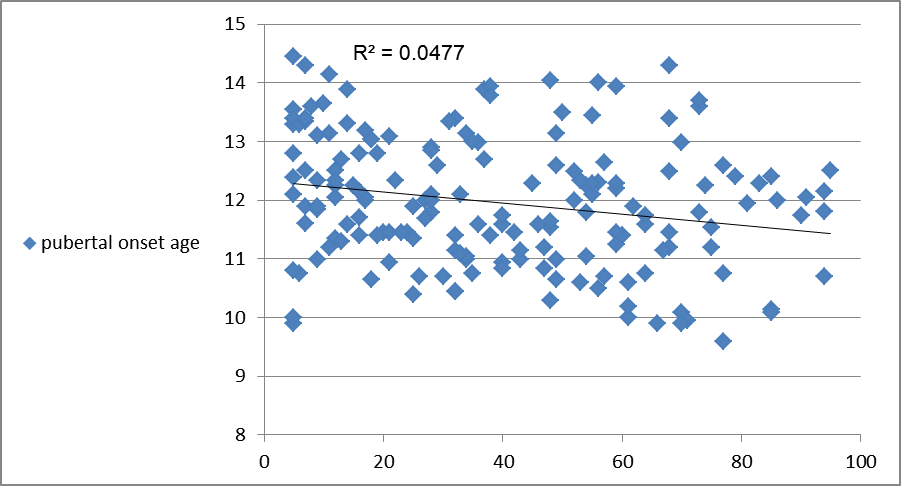
**

**Fig 2. Polish boys: Pubertal onset age vs BMI percentile.**

**
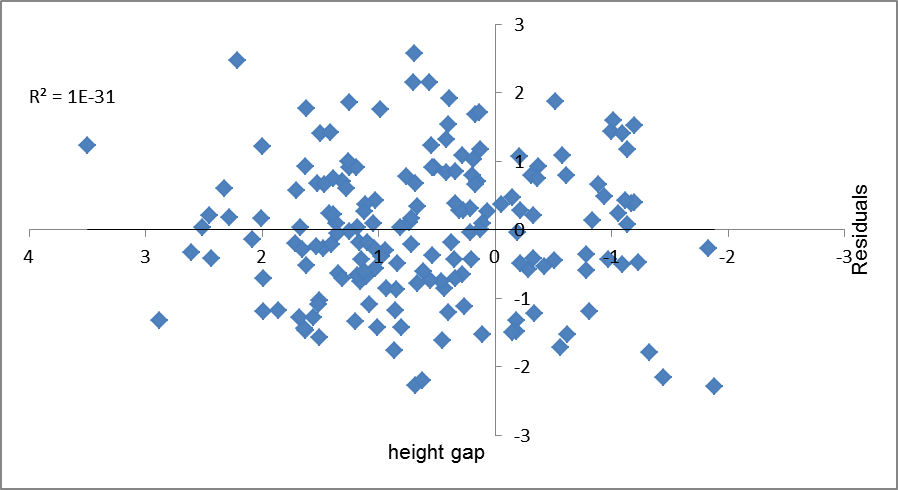
**

**Fig 3. Polish boys: Height gap residual plot.** This is the residual plot of the parameter height gap from the multivariable regression analysis of this parameter and the BMI percentile as independent parameters vs onset age of puberty as the dependent parameter.


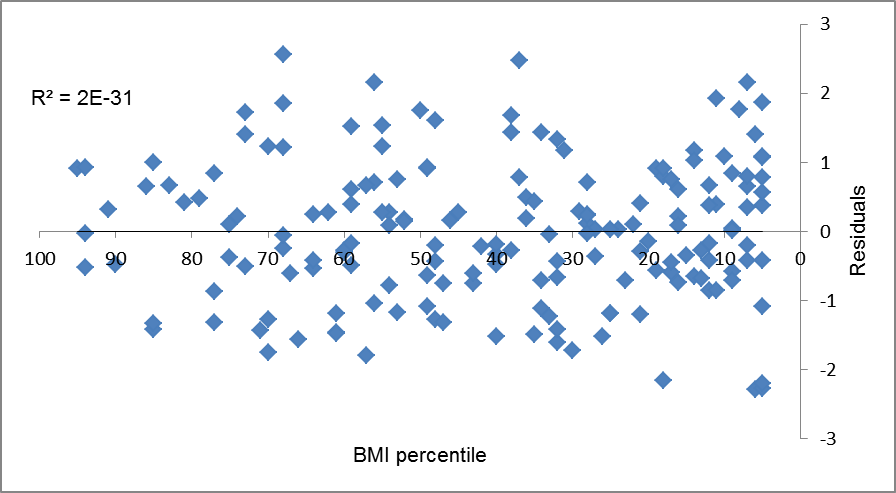


**Fig 4. Polish boys: BMI-percentile residual plot.** This is the residual plot of the parameter BMI-percentile from the multivariable regression analysis of this parameter and the height gap as independent parameters vs onset age of puberty as the dependent parameter.
